# In Defense of an Academic Career in Microbiology

**DOI:** 10.1128/mSphere.00575-17

**Published:** 2018-05-02

**Authors:** Patrick D. Schloss

**Affiliations:** a Department of Microbiology and Immunology, University of Michigan, Ann Arbor, Michigan, USA; University of Wisconsin—Madison

**Keywords:** career development, higher education, profession of microbiology

## Abstract

The rise of Quit Lit describing the myriad reasons for leaving academia and constant complaining by mentors leave many trainees with little desire for an academic career. Although there are clearly structural and social problems in academia, I feel that they are outweighed by the benefits of working and living in an academic environment. Every academic values different things about their job, and here I outline the factors that keep me in academia. To make sure that our best scientists are not scared away from academia, we must provide balance to the negativity that regularly surrounds discussions of careers in academia.

## Abstract (precis)

The rise of Quit Lit describing the myriad reasons for leaving academia and constant complaining by mentors leave many trainees with little desire for an academic career. Although there are clearly structural and social problems in academia, I feel that they are outweighed by the benefits of working and living in an academic environment.

## OPINION/HYPOTHESIS

About 10% of the proposals that I have written have been accepted for funding. I submit manuscripts expecting them to be rejected. I spend half my work hours either answering e-mails or in meetings. I have been negotiating access to a set of samples for the past 5 years with no end in sight. If I want to use an autoclave, I have to take a 3-h training module. I struggle to get a transparent report of the burndown rate on my research budgets. My value to the university appears to be linked to the amount of funding that I bring to the university. Although a paper of mine may be cited dozens of times, it is discounted because it is published in a journal with a low impact factor. My most impactful piece of work is a software package that is literally used by thousands, yet has not had direct funding for the past 5 years. In the same year that I was promoted to full professor, an indication that I have an international reputation in my field, my department told me that I needed to cultivate an international reputation. Despite several high-impact findings, I cannot pique the interest of our intellectual property or development offices. I could go on with the various pinpricks that add up to me literally smacking my forehead against my desk. My colleagues and I love to complain about our jobs. Why do we stay in academia if we are so miserable in the Ivory Tower? Because the truth is that despite all these pinpricks and our moaning, we have some pretty damn good jobs.

Unfortunately, this message is not getting to our trainees. A recent study published in *PLOS ONE* tracked 854 graduate students in STEM disciplines at 39 tier 1 institutions (1). They found that 80% of the students were initially interested in an academic career. By the time that the students graduated, 55% remained interested. The authors concluded that the decline in interest was driven by the realization that students’ initial perceptions of academic positions were not aligned with their experiences of life in academia. Many may suggest that 55% is an appropriate percentage of students who should have academic aspirations, and others may think that 55% is still too high. In fact, data from the NIH suggest that 23% of biomedical Ph.D.’s will become tenure-track faculty members (2). Indeed, just as many undergraduate students declare themselves as “premed” or “prelaw” because “smart people” become physicians and lawyers, many graduate students likely declare themselves on an academic track because that is incorrectly perceived as the pinnacle of graduate school. Indeed, there are many possible career paths for those with a Ph.D. who are underappreciated. Regardless, there is a risk that those who persist in the 55% are not the right 55%. We know that students turn away from academic careers because of implicit and structural biases against underrepresented groups. For example, among microbiologists, more than 60% of graduate students are women, yet among faculty, the number drops to below 40% for those in academic careers (3). Similar trends have been reported among minority groups. Among first-generation students, there is a difficulty in navigating the academic system and understanding the expectations and skills needed to be successful. It is critical that sources of bias shaping the composition of the professoriate are removed to ensure that the most diverse and exceptional students pursue academic careers.

I cannot speak to the very real difficulties faced by women, minorities, or first-generation graduate students. However, I can speak to the horror that comes across my trainees when they see my calendar or hear about a proposal scored in the top 13% not receiving funding. If faculty members spend large amounts of time complaining about the myriad problems that they have in their careers and little time celebrating what is great about their careers, then is it any wonder that their trainees come to realize that this is not an attractive career path? It is likely that taken out of context, the negative factors of an academic career will limit the diversity and quality of the professoriate. The life of an academic is not for everyone. Yet the attention-grabbing headlines of Quit Lit articles detailing another academic who has just had enough are rarely balanced by staypieces (4–7). We seriously risk scaring away excellent researchers from academic careers because we do not help them to see the factors that keep us in academia.

To those who have ever doubted it, I love being a professor. To be clear, I am a newly minted full professor at the University of Michigan School of Medicine, where I have minimal teaching responsibilities but significant expectations of my research output. I am also a white man who is the son of two academics and has always lived in college towns. These caveats may or may not cloud your perceptions of how my experiences relate to your own, but I think my reasons for loving my job are fairly generalizable. Let me list 10 of the reasons that I love being an academic.

### Academic freedom.

I can study anything I want as long as I can fund it. Of course, funding levels are quite low and show little likelihood of improving, so there are some constraints on what I can study. That being said, I have had funded projects related to biofuels, soil health, colon cancer, marine biology, infectious diseases, software development, and curriculum development. I have worked in Schools of Engineering, Agriculture, Life Science, and Medicine. As someone who studies microbial communities and their impacts on environmental and human health, academia has given me diverse opportunities to pursue my interests. I have never had anyone tell me to not pursue my interests. Although I have a department chair and dean, I have never felt that I have a boss or manager.

### Ownership.

Related to this last point, I have ownership over my projects. As the primary investigator on a project, I can move the project in whatever direction I want. No one is going to come to me 2 years into a 5-year project and say, “This project is floundering. Kill it.” I have considerable control over who works on a project, whom I work with, and what our outputs are. I have shunned collaborators for being a pain to work with. I have sought out projects to engage collaborators I enjoy working with. No one has ever told me whom to include in my sandbox, and I am able to set the rules.

### Leadership.

I cannot control the frustrations that I encounter on a daily basis, but I can be a force for change. Like others before me (8–10), I am dedicated to using my promotion to tenure to advocate for change. I recently received an endowment to further my research. Instead of turning all of those funds to my lab, I have decided to instead support the Software Carpentry chapter at the University of Michigan, using it as a vehicle to support women in science and to create a travel grant to help families cover childcare costs (11). Without asking anyone’s permission, I recently took on the role of being the Chair of the American Society for Microbiology Journals Board. Why? Because I was sick of sitting around complaining and listening to complaints about how screwed up peer review is and the poor quality of published research. I cannot solve all of the problems, but I can do something. I believe in the importance of leading by example and by providing service to others. Academia gives me many opportunities to exercise these types of leadership.

### Trainees.

I once interviewed for a position at a government lab and was struck by the lack of students and postdocs. The place seemed dead. Undergraduate, graduate, and postdoctoral trainees breathe life into my research. I only have so many ideas and far fewer good ideas. My research program has succeeded because these trainees expand my horizons, ask questions differently, and find new collaborators. Training the next generation of scientists well is extremely difficult. I am not a professor who tells people what to do. I try to motivate people to do what I want or what I think is best. My trainees reciprocate this by forcing me to question my assumptions. Because of the trainees who work with me, the papers we published this year are better than those from 5 years ago.

### People.

I have been privileged to meet some of the most amazing people through my career. They all see the world differently. They have different life stories, training, research interests, and personalities. Because of social media, I have been able to expand my network and find new collaborators. There are certainly some people who have made my life miserable, and I do my best to cut them out of my life. But there are far more people whom I enjoy engaging with to talk science, mentoring, and teaching. The constant turnover of trainees in a university constantly refreshes our environment. Beyond their scientific pursuits, most of our trainees are experiencing a dynamic period of their life. They are generally independent from their parents for the first time, exploring romantic relationships, having kids, and experiencing their first salaried jobs. This makes the job of mentoring them fun, but also very difficult as they navigate what they are going to do with their lives.

### Teaching.

My current appointment in a medical school does not expect me to teach much. I enjoy teaching: watching someone light up when they analyze data is a pretty special sight. Having a colleague stop me in the hall to thank me for teaching their student something that is changing how their lab functions is amazing. Here, too, I have flexibility in what I teach. Although many of us have service courses that we are expected to help teach, I also have the flexibility to teach anything else I want and as much as I want. A significant benefit of teaching is that every time I get in front of a room, I learn something about the material I am teaching.

### 20% time.

All of the institutions that I have worked in have had a policy that I can devote 20% of my effort (i.e., 1 day a week) to noncampus work. There are some caveats to this policy, but it is pretty cool to think that the university might actually encourage me to do something independent of the university. I have never used my full 20%, but in the past I have been able to use this time to teach workshops, serve as expert witness in a class action suit, and do consulting. These are not activities that will replace my research, but they do bring variety to my life and have really opened my eyes to how other people work. This is such a good idea that Google has adopted the 20% policy; Gmail was someone’s 20% project (12).

### 80% time.

There is a quip that to succeed in academia you have to work 80 h a week, but you can work any 80 h. I am not a fan of working 80 h, but I am a fan of working any 35 to 45 h I want. I can come home early to take my kids to the doctor, be there for special family events, help my kids with their 4-H projects, help my neighbor bale straw, or stay home because I feel ill. I will make up the time by working a bit longer the next day, responding to e-mails at night or over the weekend, and so forth. I have never punched a clock and have never filed a sick day. I have a very flexible work schedule.

### There is no one type of professor.

A doctorate is a degree that qualifies a person for many diverse careers. To think that there is only one type of professor is naive. Even within a university, there is tremendous variation. In the medical school, I am paid a 12-month salary but am expected to cover at least 50% of the salary by grants. Across campus, others are paid 9-month salaries and can cover the remaining 3 months on grants. I teach about 1.5 credits of classes per year. Other microbiologists across campus may teach 10 credits a year. At other institutions, there may be few expectations of research productivity, but a more significant teaching load.

### College towns.

I have lived in Ithaca, Madison, Amherst, and Ann Arbor—towns that nonacademics may not recognize, but that most academics will associate as being great college towns. I have loved living in these towns and could not imagine myself living in Boston, New York, Chicago, or San Francisco. Seeing neighbors raise a blue flag with a maize block “M” on it in anticipation of the big game signals a camaraderie that is shared by the community. Whether it’s the women’s crew team or men’s football team, I love watching college sports. There is something about them that professional sports cannot meet. I enjoy being in communities where people value higher education and lifelong learning and there is a partnership in contributing to the university’s mission, regardless of whether they work for the university.

This is not to say that these things that I love about academia are not found elsewhere, nor is this an exhaustive or even representative list. Nor is it to say that there aren't significant structural and social problems in academia. However, I suspect many of our tales of woe are told because we think the grass is greener elsewhere. For me, I am not sure where it is in fact greener. A year or so into my first tenure track position, I was contacted by a headhunter to interview for a job at Monsanto. It was amazing. Yet, as I sat on the plane heading home, I went through all of the things I loved about academia and knew that was where I belonged. I withdrew from consideration for the position the next morning. Being a lifelong academic is not for everyone, but we should do a better job of expressing the positives about our jobs and our lives, and give our trainees a more holistic impression of this life.
